# Caulerpin Delivery via Pluronic-Free Cubosomes: Unlocking the Therapeutic Potential of a Pigment from an Invasive Marine Algae

**DOI:** 10.1021/acs.molpharmaceut.5c00340

**Published:** 2025-07-01

**Authors:** Karolina Krautforst, Julita Kulbacka, Marco Fornasier, Rita Mocci, Andrea Porcheddu, Antonio Pusceddu, Davide Moccia, Sergio Murgia, Urszula Bazylińska

**Affiliations:** † Department of Chemical and Geological Sciences, 3111 University of Cagliari, s.s. 554 bivio Sestu, I-09042 Monserrato, CA, Italy; ‡ Department of Physical and Quantum Chemistry, Faculty of Chemistry, 49550 Wroclaw University of Science and Technology, Wybrzeze Wyspianskiego 27, 50-370 Wroclaw, Poland; § CSGI, Consorzio Interuniversitario per lo Sviluppo dei Sistemi a Grande Interfase, via della Lastruccia 3, 50019 Sesto Fiorentino, Florence, Italy; ∥ Department of Molecular and Cellular Biology, Faculty of Pharmacy, Wroclaw Medical University, Borowska 211 A, 50-556 Wroclaw, Poland; ⊥ Department of Immunology and Bioelectrochemistry, State Research Institute Centre for Innovative Medicine Santariškių g. 5, LT-08406 Vilnius, Lithuania; # Division of Physical Chemistry, Department of Chemistry, 5193 Lund University, SE-22100 Lund, Sweden; ∇ Department of Life and Environmental Sciences, University of Cagliari, Cittadella Universitaria Monserrato, S.P. 8 Km 0.700, I-09042 Monserrato, CA, Italy

**Keywords:** algae, caulerpin, anticancer, cubosomes, nanocarriers, pancreatic tumor

## Abstract

Pancreatic cancer remains one of the deadliest cancers due to its resistance to conventional therapies, necessitating the development of novel treatment strategies. This study investigates the anticancer potential of caulerpin, a bisindole alkaloid derived from the invasive marine alga *Caulerpa cylindracea*, encapsulated in biocompatible cubosomes. Caulerpin was sustainably extracted via microwave-assisted methods and formulated into lipid-based bicontinuous cubic liquid crystalline nanoparticles using Pluronic-free surfactants (sodium taurocholate and Span 80), resulting in high encapsulation efficiency and structural stability at physiological temperature (37 °C). The formulation included cubosomes coexisting with L3 sponge nanoparticles and vesicles. *In vitro* studies on BxPC-3 pancreatic cancer cells demonstrated that encapsulated caulerpin significantly outperformed the free compound, inducing marked apoptotic features such as cytoskeletal disruption and cell shrinkage, as confirmed by holotomographic microscopy and F-actin bioimaging. The enhanced therapeutic efficacy is attributed to the improved protection and sustained intracellular availability of encapsulated caulerpin, which is not rapidly metabolized as in its free form. These findings suggest that caulerpin-loaded cubosomes may represent a promising nanotechnology-based strategy for pancreatic cancer treatment.

## Graphical abstract

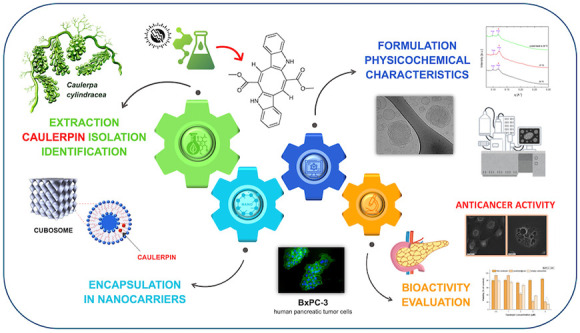

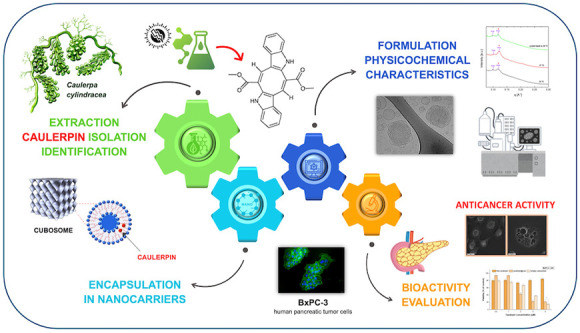

## 1 Introduction

Green marine algae, genus *Caulerpa* constitutes a valuable source of different secondary metabolites like alkaloids, terpenoids, flavonoids, steroids, and tannins with potential bioactive properties against many diseases, therein anticancer activity by targeting and modulating multiple cancer cell-specific signaling pathways and cascades.^1,2^ These metabolites have been shown to affect mitochondrial health and various metabolic and stress pathways through interaction with signaling proteins such as AMPK, PTP1B, or HIF-1, as well as cell cycle progression.^1,3^ One of them, caulerpin, an orange-red pigment characteristic for *Caulerpa* species, was found in *Caulerpa cupressoides* (*C. cupressoides*), *C. paspaloides*, *C. prolifera*, *C. sertularioides*, and *C. cylindracea*.^4–6^ Caulerpin’s chemical structure was first elucidated by Aguilar-Santos in 1970 ^7^ and later revised by Maiti and Thomson in 1977 ^8^ and consists of two indole groups separated by a central cyclooctatetraene ring with two methyl ester groups.^1,9^ This specific structure classifies caulerpin into the group of natural bisindole alkaloids, characterized by two indole units, more potent concerning their biological activity than their corresponding monomeric units.^10^ Various naturally derived medicinal products are based on indolic alkaloids, like the most studied vincamine, vincristine, or vindesine mainly due to their activities as cerebral vasodilators, antiarrhythmics, antihypertensives, and anticancer agents.^11,12^ Caulerpin has been reported to exhibit a range of biological activities, including antinociceptive and anti-inflammatory effects,^5^ as well as antimicrobial,^9^ antiviral,^13^ and anticancer properties.^4,14^

Pancreatic cancer is one of the deadliest malignancies due to its special complex tumor microenvironment, as well as the multitude of mechanisms and pathways responsible for the exceptional resistance of this cancer to treatment.^15–19^ Attention should also be paid to the prevention of this type of disease, as it may be associated with inflammatory processes in the pancreas caused by using certain drugs. Patients with type 2 diabetes may be particularly susceptible, since recent work by Li et al. proved that the use of antidiabetic agents from the SGLT2 inhibitor (SGLT-2i) class has been linked to cases of acute pancreatitis (AP).^20^ AP, especially when recurrent or progressing to chronic pancreatitis, is a known risk factor for the development of pancreatic cancer.^21^ Therefore, monitoring patients who experience drug-induced AP (e.g., those related to SGLT-2i use) may be crucial for the early detection of neoplastic changes in the pancreas.

Xu et al. in 2019 reported that inhibition of PTP1B protein blocks pancreatic cancer progression by targeting the PKM2/AMPK/mTOC1 pathway.^22^ It was proved that caulerpin presents PTP1B inhibitory activity, therefore indicating its potential influence on pancreatic cancer progression.^23,24^ Moreover, caulerpin is known to induce mitochondrial dysfunctions, thereby disrupting the crucial energy balance of cells.^25^ In 2017, Yu et al. demonstrated on SW480 and LoVo cells (colorectal cancer) that this effect is caused by metabolic reprogramming and AMPKα1 pathway activation: caulerpin inhibits complex I of a mitochondrial electron transport chain, accompanied by the dissipation of mitochondrial membrane potential, and decreases oxygen consumption rate in cancer cells with a simultaneous surge of reactive oxygen species (ROS) and decrease in ATP production.^14^ In the central regulatory node of the energy metabolism and tumor progression, there is adenosine monophosphate (AMP)-activated protein kinase (AMPK), known for the detection of high ADP or AMP levels, indicating the depletion of energy stores.^26^ Therefore, in response to the increase in the AMP/ATP ratio, high expression of AMPK energy sensor was proved to be activated by caulerpin anticancer treatment.^14^ On the other hand, a novel AMPK inhibitor developed recently by Schneider et al. presented an increase in the sensitivity of pancreatic cancer cells to the induction of ferroptosis—a specific type of programmed cell death dependent on iron and oxidative stress, leading to damage of cell membranes.^27^ The unfavorable prognosis of pancreatic ductal adenocarcinoma is largely due to a hypoxia-driven tumor microenvironment regulated by carbonic anhydrase IX (CAIX), which, as demonstrated by Ghosalkar et al., can be targeted with methazolamide to enhance the sensitivity of therapy-resistant PDAC cells through inhibition of CAIX.^28^ Moreover, Liu et al. proved that caulerpin also presents hypoxia-inducible transcription factor (HIF-1) inhibitory activity, by inhibition of the mitochondrial electron transport to complex III.^3^ Considering the vast amount of work on the potential anticancer activity of caulerpin, it is noteworthy that out of the many various cancer cell lines already studied, such as colorectal cancer^4,14^ breast cancer,^3^ or ovarian cancer,^25^ there is still no research on the caulerpin’s activity against pancreatic cancer.

It has been proven that *C. cylindracea* Sonder 1845 is one of the most invasive species in the Mediterranean Sea,^29^ rapidly spreading and causing many ecological changes and reducing biodiversity at a range of spatial scales.^30–34^ This alga can alter the native species’ behavior, with alleged adverse repercussions even on fish growth patterns, behavior, and dynamics of the population.^35^ Therefore, the management of the invasive algae biomass to isolate bioactive compounds could constitute an economical and widely available pharmaceutical source from natural biomass and at the same time an environmentally friendly solution, regulating the marine environment. Recent trends indicate increased application of innovative green extraction methods, including microwave-assisted extraction (MAE), characterized by increased efficiency, reduced time and lowered process costs.^36^ Cantarino et al. proved that MAE is a suitable and efficient method for caulerpin extraction with high yield from these algae.^37^ Conversely, current methods for caulerpin synthesis generally results in low yields (<50%) from indole.^38–40^ These methods require at least three synthetic steps, some involving reflux conditions and the isolation of intermediates. Moreover, they rely on toxic reagents, such as POCl_3_ and piperidine. In this context, extracting the bis-indole alkaloid directly from renewable biomass offers a significant advantage, enhancing both sustainability and efficiency. However, the low solubility and instability of caulerpin in aqueous media, associated with its hydrophobic nature, limit its use in the cellular environment due to reduced bioavailability.^41^ Therefore, there is a significant need to circumvent this problem, and encapsulation in the appropriate nanocarriers appears to be a potential solution. There are many innovative combinatorial approaches to novel anticancer treatments, especially in the field of various nanosystems. Nie et al. developed metal organic framework coated MnO_2_ nanosheets for co-delivery of a survivin silencing RNA-cleaving DNAzyme self-activated by Mn^2+^, and antineoplastic doxorubicin, with the additional ROS generation by Mn^2+^, for efficient chemo–gene combinatorial treatment of cancer proved by *in vitro* and *in vivo* studies.^42^ Furthermore, recent studies of Feng et al. on tailoring the molecular structure of doxorubicin carrier-free prodrug nanoassembly by linking with fatty alcohols have shown that the nanosystem modified with long-chain fatty alcohol improved stability but slowed the disassembly and drug release process.^43^ On the other hand, prolonged circulation in the body and tumor accumulation were proved by hexadecanol-modified doxorubicin prodrug and significantly enhanced antitumor activity *in vivo*.^43^

The colloidal dispersion of bicontinuous cubic lipid-based lyotropic liquid crystalline nanoparticles in water with the use of suitable surfactants and stabilizers results in nanostructured systems, referred to as “cubosomes”, having sizes ranging from 100 to 400 nm.^44,45^ These honeycomb-like structures comprise curved bicontinuous lipid bilayers organized in three dimensions, forming two internal not interconnected water channels, that can be exploited by various bioactive ingredients.^46–48^ Cubosomes are able to deliver bioactive cargo to the target disease tissue, protecting it from the degrading cellular environment and, in the case of water-insoluble compounds, preventing their aggregation.^49^ Recently published works^50–53^ indicate that cubosomes are gaining utility and acceptance as anticancer drug delivery systems with possible theranostic approaches. For the potential treatment of cervical cancer, Zhang et al. proposed combinatorial cubosomes coloaded with well-known anticancer drugs: cisplatin and paclitaxel and coated with poly-ε-lysine for sustained drug release, with an enhanced therapeutic efficacy proved against HeLa cells.^52^ Moreover, cubosomes loaded with copper acetylacetonate as anticancer drug, were for the first time functionalized with Affimer proteins to actively target carcinoembryonic antigens on LS174T colorectal cancer cells.^54^ In this way, Pramanik et al. have developed novel promising Affimer-tagged cubosomes by their first comprehensive preclinical evaluation through *in vitro* and *in vivo* experiments, proving selective accumulation in cancer cells in comparison to normal ones, minimal tissue absorption in vital organs with no significant signs of toxicity, along with the reduced tumor growth.^54^ Such examples demonstrate an enormous therapeutic potential of these liquid crystalline nanocarriers to fight various types of cancer; therefore, research on them should be continuously expanded and updated.

With the above knowledge, we aimed to assess the antitumor potential of caulerpin, isolated from *Caulerpa cylindracea* using green MAE and chromatographic methods against highly resistant pancreatic cancer cells (BxPC-3). To overcome the challenges posed by the tumor microenvironment, which often impedes drug efficacy, we propose encapsulating caulerpin within newly formulated cubosomes. These cubosomes (caulerpin@cub) offer enhanced biocompatibility and stability, achieved through an optimized surfactant mixture instead of traditional Pluronic copolymers.^55^ The combination of sustainable caulerpin extraction from *C. cylindracea* biomass and its encapsulation in advanced biocompatible nanocarriers hold promise for improving the efficacy of this potential anticancer agent against extremely resistant pancreatic cancer.

## 2 Experimental Section

### 2.1 Materials

The main building block used for cubosomes preparation was glycerol monooleate (1-monooleoylglycerol, MO, RYLO MG 19 PHARMA, 98.1 wt %), kindly provided by Danisco A/S (Denmark). Other components of the cubosomes were Span 80 (Sp80, sorbitan monooleate, steric stabilizer) and taurocholic acid sodium salt hydrate (TC, taurocholate, bile salt) purchased from Sigma-Aldrich (Germany). The solvents used in the extraction and caulerpin isolation process (ethanol, hexane, diethyl ether, and ethyl acetate) were purchased from Honeywell (Germany). Anhydrous sodium sulfate as a drying agent was purchased from Sigma-Aldrich (Italy). The chromatographic column was performed using EcoChromeTM MP Silica gel 60 Å, particle size 0.040–0.063 mm (230–400 mesh), purchase from Merck (Germany). Cerium sulfate used in TLC analysis was purchased from Merck (Germany).

Deuterated chloroform used in the NMR analysis was purchased from Sigma-Aldrich (Germany). Water was freshly distilled and filtered through a Milli-Q apparatus (Millipore, Milan, Italy) and is referred to as water (W). For the preparation of the cubosomes, water was filtered (before the preparation) with a 0.22 μm pore size hydrophilic filter (Milli-Q system, Millipore, Germany).

### 2.2 Biomass Collection and Preparation

Fresh *C. cylindracea* thalli were collected from the Mediterranean Sea in Sardinia (Italy). The algae were washed with water to remove sand, salt, and epiphytes, deep-frozen at −80 °C, and freeze-dried (Benchtop, VirTis, US) for 48 h (−51.8 °C, 0.150 mbar). The dry weight (DW) of the algae was 4.3 ± 0.4% of the fresh biomass. Then, the freeze-dried algae were milled into a powder with the use of a ball-milling technique to increase the extraction surface. For this purpose, the freeze-dried *C. cylindracea* (1.5 g) was loaded into a zirconium oxide grinding jar (35 mL) equipped with 2 balls (ϕ = 8 mm). The jar was sealed and shaken for 15 min at a frequency of 20 Hz using a VWR Beater Mixer Mill apparatus. The powdered biomass was then stored in the refrigerator and used in the next steps.

### 2.3 Caulerpin Isolation

Caulerpin was isolated from the powdered biomass of *C. cylindracea* marine algae as follows (Figure 1). The extraction was performed in a two-step process including solid–liquid microwave-assisted extraction and liquid–liquid extraction with pigment fractionation in a separatory funnel. Then, caulerpin was isolated and purified on a silica chromatographic column and identified by UV–vis spectrophotometry and ^1^H NMR spectroscopy.

**Figure 1:**
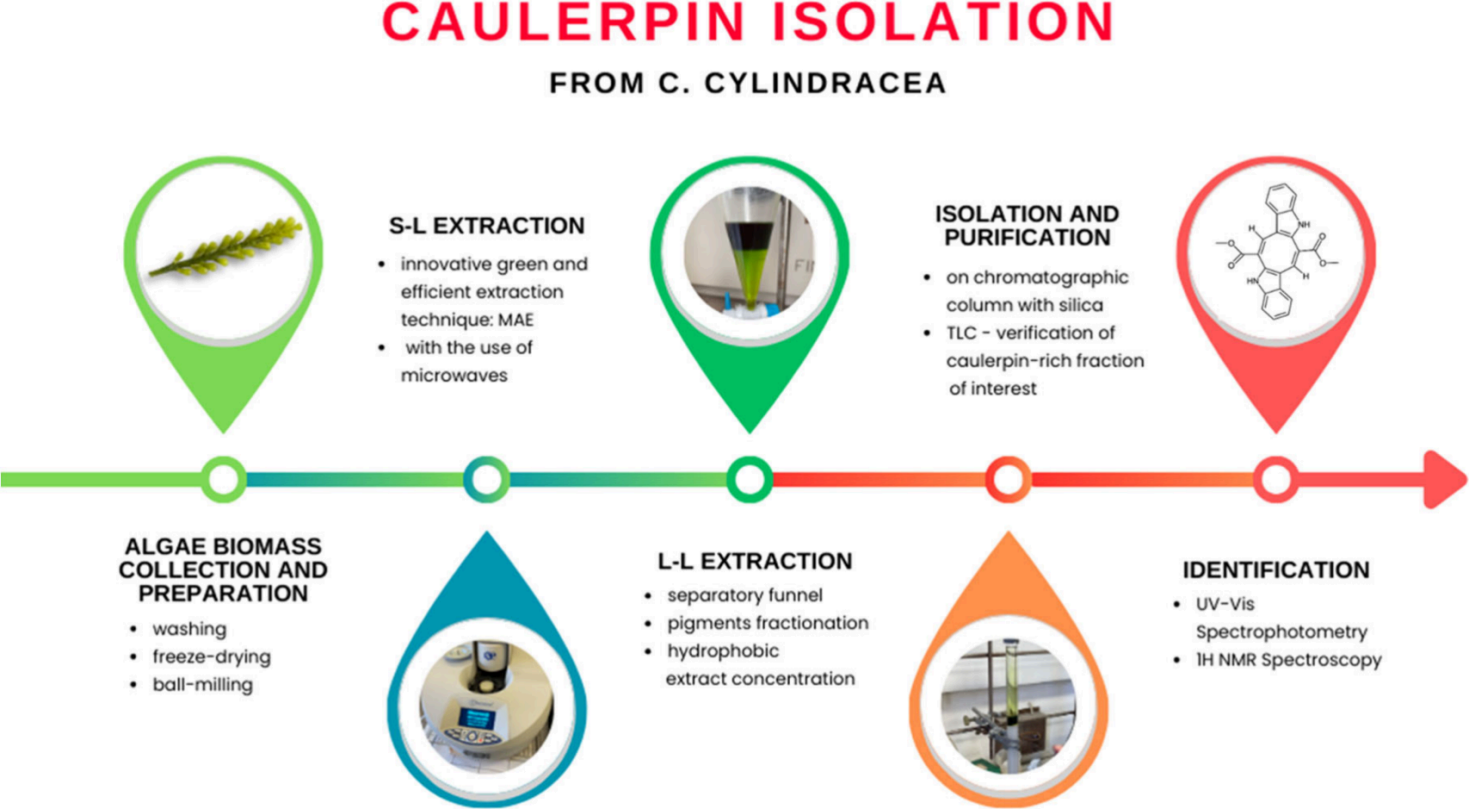
General scheme of the path from *C. cylindracea* algae biomass collection and preparation to pure caulerpin isolation and identification.

#### 2.3.1 Microwave-Assisted Extraction

To release bioactive compounds from the cell wall into the solvent solution, solid–liquid extraction was performed using the MAE technique (Discover SP, CEM). Ethanol was chosen as a solvent (in a solid–liquid ratio of 0.1 g of dry algae/mL of solvent) since it is a green solvent and gives high extraction efficiency.^56^ The biomass was subjected to microwave-assisted extraction with stirring at 90 °C for 7 min with a power of 200 W and pressure of 300 PSI. Extraction conditions were chosen based on literature data concerning the evaluation of the most efficient extraction methodology for caulerpin isolation.^37^ After that, the solution was centrifugated (4000 rpm/10 min) (REMI R-9M, Remi Elektrotechnik Ltd., Indie) and the supernatant was evaporated and dissolved in diethyl ether for further liquid–liquid extraction in a separatory funnel 1:1 with distilled water. The organic diethyl ether phase, rich in hydrophobic pigments of interest, i.e., caulerpin, was collected, dried with anhydrous sodium sulfate, and concentrated under reduced pressure in a rotavapor (BÜCHI R-200, BÜCHI Labortechnik AG, Switzerland) giving the final crude *C. cylindracea* extract used in the next steps.

#### 2.3.2 Isolation of Caulerpin from *C. cylindracea* Extract and Purification on Chromatographic Column

Silica gel was loaded into the chromatographic column and conditioned with a mixture of hexane:ethyl acetate mixture (8:2). The crude *C. cylindracea* extract was loaded into the chromatographic column by dry-loading and by adsorption on silica gel before dissolution in diethyl ether. The column was then eluted with a solution of hexane:ethyl acetate (8:2). The fractions were collected until the caulerpin was found by TLC identification (hexane:diethyl ether 1:1) based on characteristic reaction with cerium sulfate (resulting in a red spot after gentle heating) and literature data of the *R_f_* parameter.^25^ Caulerpin-rich fractions of interest were collected, and the solvents were evaporated in a rotavapor. The resulting orange-red solid was further purified by crystallization from acetone and hexane.

#### 2.3.3 Caulerpin Identification by UV–vis Spectrophotometry and ^1^H NMR Spectroscopy

To identify the isolated compound, its absorbance spectrum in ethanol was recorded using a UV–vis spectrophotometer (Cary Series UV–vis–NIR spectrophotometer, Agilent Technologies, U.K.) using a quartz cuvette (optical path, 1 cm) as well as ^1^H NMR spectrum in CDCl_3_ to identify its chemical structure and purity. ^1^H NMR measurements were carried out at 25 °C using a Bruker Avance 300 MHz (7.05 T) spectrometer at the operating frequency of 300.131 MHz. A standard BVT 3000 variable-temperature control unit with an accuracy of 0.5 °C was used.

### 2.4 Caulerpin Encapsulation in Cubosomes

Cubosome formulations were prepared by following a previous methodology from our group.^55^ In the beginning, MO was melted at 40 °C and caulerpin added and mixed with the lipid phase until obtaining a homogeneous mixture. After that, a solution of TC and Sp80 in water was added to the lipid phase and immediately sonicated by an ultrasonic processor (UP100H, Hielscher Ultrasonics, Germany) in 2 cycles (2 and 3 min) with 90% amplitude with the pulse ON for 1 s followed by 1 s of break. The empty cubosomes were prepared in the same manner without the addition of caulerpin. The composition (wt %) of empty and loaded (caulerpin@cub) cubosomes was respectively MO:TC:Sp80:W=3.5:0.2:0.1:96.2.

### 2.5 Dialysis and Encapsulation Efficiency

In order to remove the excess cargo after the encapsulation in nanocarriers, caulerpin@cub formulation was dialyzed after loading it into a tubing cellulose membrane (14 kDa cutoff; Sigma-Aldrich), in 2 L of water for 2 h at room temperature, changing the water after 1 h.

The encapsulation efficiency (EE) was evaluated by UV–vis spectroscopy. The absorption spectra were acquired using a UV–vis spectrophotometer (Cary Series UV–vis–NIR spectrophotometer, Agilent Technologies, U.K.) using a quartz cuvette (optical path, 1 cm). The standard calibration curve of caulerpin was prepared to calculate the weight of the encapsulated caulerpin before and after dialysis, with the data fitted to a linear regression (*R* ^2^ = 0.9999). Absorption spectra of the studied cubosome formulations before and after dialysis were measured after dissolving in ethanol (dilution 1:5, v/v), and the EE was calculated through the following expression:

$$\mathrm{EE}\left(\%\right)=\frac{\mathrm{weight of the caulerpin after dialysis}}{\mathrm{weight of the caulerpin before dialysis}}\times 100$$

### 2.6 Small-Angle X-ray Scattering (SAXS)

The structure assessment of caulerpin@cub formulation was performed by small-angle X-ray scattering (SAXS) experiments at the SAXSLab instrument (JJ-Xray, Denmark), following our previously published protocol.^57,58^ For this purpose, the samples were placed in quartz capillaries and measured at a sample to detector distance of 360 mm for around 2 h per sample at a given temperature. The intercrystalline spacing (*d*) was calculated after evaluating the *q* positions of each peak in the SAXS patterns using the following equation:

1 $$d=\frac{2\pi }{q_{\mathrm{peak}}}$$

Then, the lattice parameter (*a*), describing the crystalline unit of the inverse bicontinuous cubic phases, was extrapolated from eq 2:

2 $$a=d\sqrt{h^{2}+k^{2}+l^{2}}$$

where *h*, *k*, and *l* are the Miller’s indexes. Finally, the water channel radii of the cubosome and sponge nanoparticles were evaluated by applying eqs 3 and 4, respectively:

3 $$r_{w}=\left(a-l\right)\sqrt{\frac{A_{0}}{-2\pi \chi }}$$

4 $$r_{w-L3}=\frac{d_{L3}}{d_{\mathrm{cubic}}}r_{w-\mathrm{cubic}}$$

where χ and *A*_0_ are, respectively, the Euler characteristic and the surface area of the specific geometry (Pn3m, χ = – 2, and *A*_0_ = 1.919), and *l* is the MO hydrophobic chain length at 25 °C (17 Å).^59^

### 2.7 Dynamic and Electrophoretic Light Scattering (DLS, ELS)

Size, polydispersity index, and ζ-potential of the nanoparticles were evaluated by dynamic and electrophoretic light scattering (DLS and ELS, respectively). All measurements were conducted in triplicate on the diluted (1:50, v/v) samples at 25 and 37 °C on a ZetaSizer Nano ZS by Malvern Instrument (Malvern, U.K.) (backscattering angle, 173°; with a 4 mW He–Ne laser at 632.8 nm). The apparent hydrodynamic radius (D_h_) and polydispersity index (PdI) were extracted by a second-order cumulant analysis of the autocorrelation curves of each sample. The ζ-potential was evaluated by ELS measurement and application of the Smoluchowski equation to the electrophoretic mobility. The signal was collected at a fixed angle of 17°.

### 2.8 Cryogenic Transmission Electron Microscopy (cryo-TEM)

The morphology of the caulerpin@cub formulation was imaged by cryogenic transmission electron microscopy (cryo-TEM). Briefly, 4 μL of the studied sample was blotted on a carbon-coated copper grid at 25 °C with a relative humidity of 90%, within 24 h from preparations, and then imaged using a JEOL JEM-2200FS transmission electron microscope (JEOL, Tokyo, Japan).

### 2.9 Biological Activity

#### 2.9.1 Cell Lines Culturing

The BxPC-3 cells (human pancreatic cancer cell line, ATCC, CRL-1687) were maintained in culture flasks with a surface area equal to 75 cm^2^ (Falcon cell culture flasks) in RPMI 1640 (Sigma-Aldrich, Poznan, Poland) supplemented with 10% fetal bovine serum (FBS) and 50 μg/mL penicillin and streptomycin (Sigma-Aldrich, Poznan, Poland). The cultures were incubated in a humidified atmosphere with 5% CO_2_ at 37 °C. The cells used for all experiments were detached by trypsinization (Trypsin–EDTA solution) and neutralized with Dulbecco’s phosphate buffered saline (DPBS), which was purchased from Sigma-Aldrich (Poznan, Poland).

#### 2.9.2 Cytotoxicity Studies

The cytotoxicity MTT (3-(4,5-dimethylthiazol-2-yl)-2,5-diphenyl tetrazolium bromide) cell proliferation assay (Sigma-Aldrich, Poznan, Poland) was performed for various caulerpin concentrations (0.5, 1, 2, 3, and 4 μM). All formulations were uniformly diluted to ensure a fair comparison between empty and loaded nanocarriers at the same MO concentration (corresponding to 56, 112, 223, 334, and 445 μg/mL) for each caulerpin concentration.

BxPC-3 cells were studied after 4, 8, 24, and 48 h of incubation. Generally, 200 μL of cells (approximately 2 × 10^5^ cells) were placed into 96-well plates (Sarstedt, Equimed, Wroclaw, Poland). Then, the absorbance was measured at 560 nm using a GloMax Discover microplate reader (Promega), which allowed evaluation of the cell viability in each group expressed as percentages of the control cells (untreated with studied samples). Each data set represents three independent biological experiments with three technical wells per condition. The data are reported as average value ± standard deviation (SD).

#### 2.9.3 Optical Microscopy (Culture Morphology)

The cell culture morphology was studied with an optical inverted microscope DMi1 (Leica Microsystems, Germany). Pictures were taken after 8 and 24 h of BxPC-3 incubation in RPMI culture medium at 37 °C. BxPC-3 cells treated with free caulerpin (2 μM), caulerpin@cub (caulerpin content = 2 μM), or empty cubosomes, and compared to untreated control cells. BxPC-3 cells were cultured on a special microscope glass.

#### 2.9.4 Holotomographic Microscopy (HTM)

Morphological changes of BxPC-3 tumor cells over time (4, 8, and 24 h) were assessed using a holotomographic microscope (Nanolive, Switzerland) by comparing control cells with 3 studied samples: cells treated with free caulerpin (2 μM), caulerpin@cub (caulerpin content equal to 2 μM), or empty cubosomes. Cells were previously detached from cell culture flasks and cultured on 35 mm^2^ Petri dishes with special microscope glass bottom (μ-Dish 35 mm, low glass bottom, Ibidi, Germany), by incubation for 24 h at 37 °C in RPMI culture medium. All microscopic preparations were viewed intravitally.

#### 2.9.5 Immunofluorescent F-Actin Staining Protocol

The integrity of the cytoskeleton was determined after 24 h of postencapsulated potential drug exposure by immunofluorescent labeling of F-actin cells with Alexa Fluor546 Phalloidin (Thermo Fisher Scientific Inc.). Fluorshield with a fluorescent DNA-binding dye of cells grown in the culture (4,6-diamidino-2-phenylindole, DAPI) was used to visualize the nuclei and mount the cells after excitation at 405 nm. Samples were examined on an Olympus BX53 fluorescence microscope (Evident Europe GmbH, Warsaw, Poland).

#### 2.9.6 Statistical Analysis

Results of the *in vitro* experiments are presented as mean values ± SD for a minimum of 3 and compared by two-way ANOVA for multiple comparisons and α = 0.05. Comparisons of samples exhibiting *p* values ≤ 0.05 and ≤ 0.01 were considered statistically significant and highly significant, respectively. In the case of viability assay, all results were compared to untreated control. Results were analyzed using the commercial software GraphPad Prism 7.0.

## 3 Results and Discussion

### 3.1 Characterization of Caulerpin and Encapsulation in Cubosomes

This study aims to evaluate the bioactivity against human pancreatic cancer cells of an alkaloid with potential anticancer properties, known as caulerpin, characteristic of several species within the genus *Caulerpa*, a group of green algae. Here, pure caulerpin was extracted from

*C. cylindracea* and encapsulated within cubosomes to improve its bioavailability and potentially increase its effectiveness in pancreatic cancer treatment.

First, the hydrophobic dark green extract from *C. cylindracea* (see Section 2.3.1) was obtained with a yield of 3.1 ± 0.3% DW. Then, after further purification process on a chromatographic column, pure caulerpin was isolated in a form of orange-red solid pigment with a yield of 0.47 ± 0.02% DW. According to the literature, this value is significantly higher than those obtained through classical methods, such as 0.1% DW of caulerpin extracted from *C. racemosa* via maceration,^60^ or 0.2% DW achieved by traditional Soxhlet extraction.^61^

Caulerpin was identified by UV–vis spectrophotometry because it is a pigment that exhibits high absorbance at characteristic ranges of wavelengths: 222, 270, 292, and 317 nm.^8^ The absorbance spectrum of the isolated compound is presented in Figure 2. The sample was then investigated by ^1^H NMR spectroscopy to further identify the product and confirm its purity (Supporting Information Figure S1). The pure caulerpin was subsequently encapsulated in cubosomes (caulerpin@cub) at a concentration of 0.125 mg/mL (EE = 92.6 ± 0.9%).

**Figure 2:**
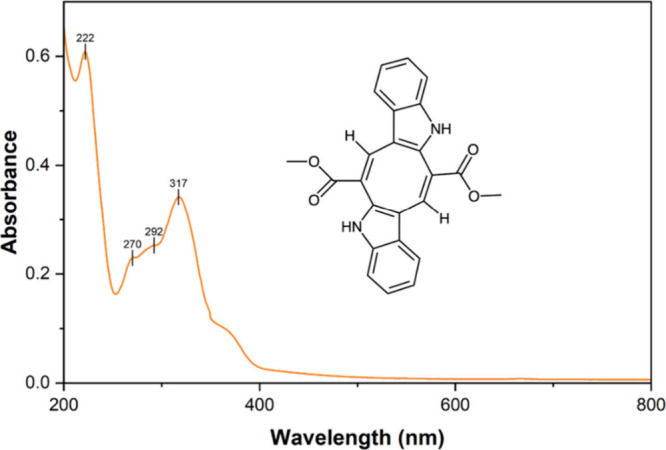
UV–vis absorbance spectrum of isolated caulerpin in ethanol exhibiting characteristic peaks at 222, 270, 292, and 317 nm along with the chemical structure of caulerpin.

### 3.2 Physicochemical Characterization of the Nanocarriers Loaded with Caulerpin

SAXS measurements were performed to evaluate the inner structure and phase of the formulation loaded with caulerpin and at different temperatures to observe possible changes in the structure of the formulation and evaluate their behavior in storage (room temperature) and potential in vivo conditions (body temperature).

The sample was then cooled to 25 °C to observe any structural hysteresis. Figure 3A presents the SAXS diffractograms of the cubosomes loaded with caulerpin, whereas Table 1 reports on the evaluated structure parameters, e.g., lattice parameter (*a*) and water channel radius (*r_w_*).

**Figure 3:**
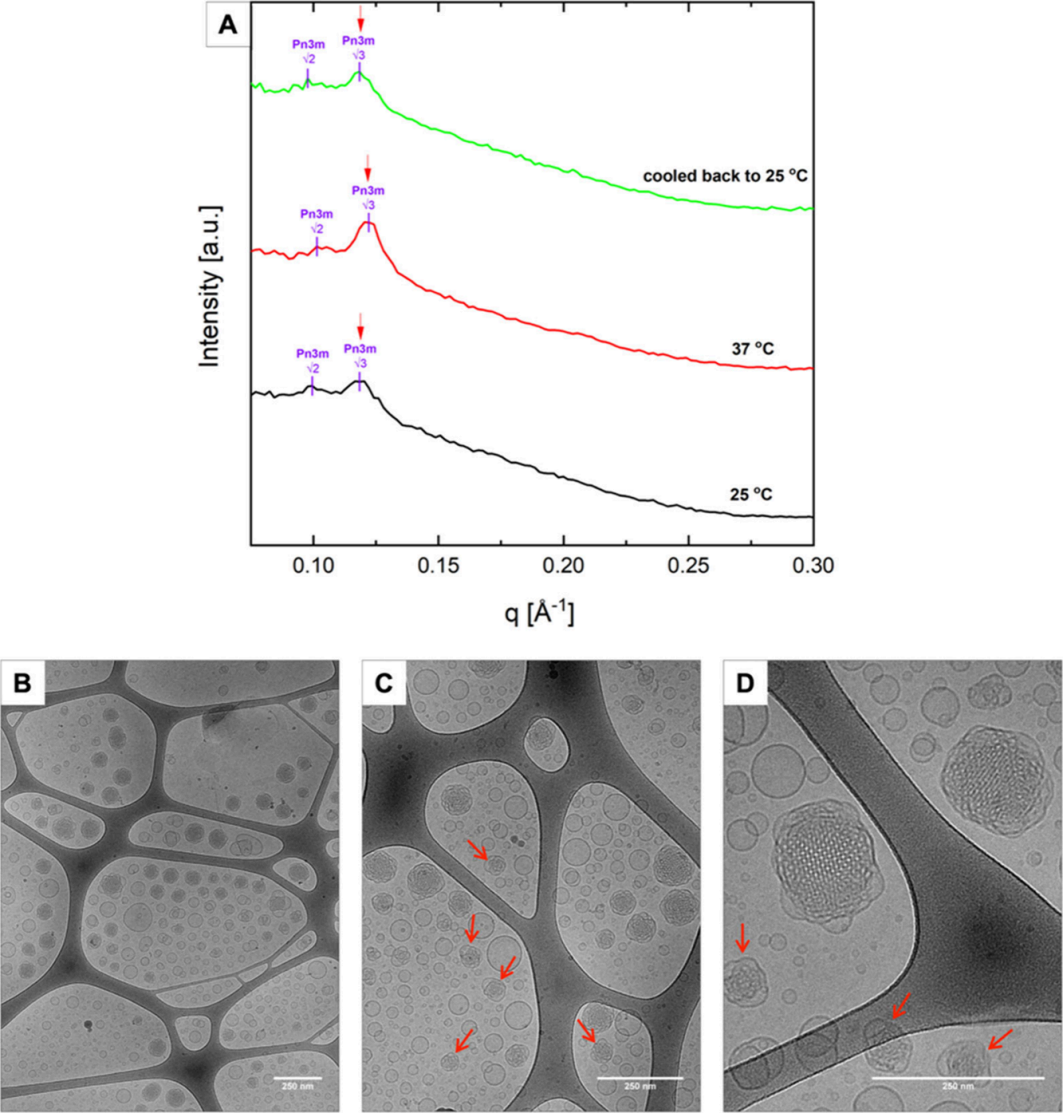
SAXS diffractograms at 25 °C, 37 °C after the heating cycle and returning to 25 °C (A) and cryo-TEM images (B, C, D) of the caulerpin@cub formulation. Red arrows indicate the presence of sponge phase (L3) nanoparticles in the formulation. The scale bar is 250 nm.

**Table 1:** Phase, Lattice Parameter (*a*), and Water Channel Radius (*r_w_*) of caulerpin@cub and Empty Cubosomes at 25, 37, and Cooled Back to 25 °C^a^

sample	T (°C)	phase	*a* (Å)	*r*_*w*_(Å)
caulerpin@cub	25	Pn3m	91 ± 1	18.4 ± 0.7
		L3		15.5
	37	Pn3m	88 ± 1	17.5 ± 0.4
		L3		14.6
	back to 25	Pn3m	91.5 ± 0.6	18.7 ± 0.2
		L3		15.4
empty cubosomes^55^	25	Pn3m	92.1 ± 0.1	22.0 ± 0.3

a Data are reported as mean ± SD.

In Figure 3A the main peaks suggesting the *Pn*3*m* cubic phase present low intensity with a high and broad diffusive band related to the presence of other particles in the studied formulation, like polydisperse vesicles. This conclusion is based on the compatibility with cryo-TEM images of this formulation presented in Figures 3B–D, discussed in more detail in the next paragraph. The two visible peaks are in the position corresponding to the Pn3m phase with the lattice parameter 90.6 ± 1.9 Å at 25 °C, a value typical for this type of cubic phase. Furthermore, as scattering is a probabilistic event and normally the peaks decrease with the order of scattering, the second peak, being more intense than the first one, indicates the possible presence of a second phase. Moreover, Sp80 tunes the packing parameter of the lipid (MO), rendering a more disordered cubic phase, commonly referred to as L3 phase (sponge phase).^62^ The first suspected peak of the L3 phase is highlighted in Figure 3A with a red arrow. Therefore, caulerpin encapsulation and the presence of Sp80 affected the structure of the nanoparticles, by changing the effective packing parameter of MO. Increasing the temperature to 37 °C causes a small shift toward higher *q* values, indicating lower values of the lattice parameter (*a*) and water channel radius (*r_w_*) of caulerpin@cub, as reported in Table 1. The shift at 37 °C is attributed to increased thermal motion of the hydrophobic tails of MO, leading to an expansion of its hydrophobic region. Consequently, this results in a reduced lattice parameter and water channel radius while maintaining the same nanostructure.^59^ Moreover, heating to 37 °C makes the first peak of the L3 phase more pronounced and the first peak of Pn3m broader, suggesting that a larger portion of the Pn3m phase transitions to L3 as the temperature increases. Nevertheless, these are speculations based on the analysis of one peak. Deeper investigations at higher resolutions or at a source with higher brilliance would be needed for complete assessment. Overall, the sample remains stable at the human body temperature of 37 °C, demonstrating its potential for *in vivo* applications.

In order to investigate the nanoparticles’ morphology, cryo-TEM measurements were performed. Panels B–D of Figure 3 are representative images of caulerpin@cub. Starting from Figure 3B, a lot of cubosome showing their typical honeycomb-like structure are observed^47^ in coexistence with other objects. In agreement with the empty formulation,^55^ the presence of vesicles is probably related to Sp80 and TC in the composition—vesicle forming ingredients.^58^ Moreover, sponge nanoparticles are observed in the sample and highlighted with red arrows (Figure 3C,D). Their presence is attributed to the formulation’s composition, as discussed in detail in the previous SAXS section. The coexistence of the bicontinuous cubic phase with the L3 phase in the formulation loaded with *C. cylindracea* algae pigment indicates the potential influence of hydrophobic cargo on the nanoparticles structure.

Then, caulerpin@cub was studied for the particles’ size described by apparent hydrodynamic diameter (D_h_), polydispersity index (PdI), and ζ-potential by DLS and ELS measurements in two different temperatures to evaluate a sample’s stability and further application *in vivo*. The results are presented in Table 2. All parameters are in line with the empty formulation characterized by the same composition (MO:TC:Sp80:W).^55^ To the best of our knowledge, only a few scientific studies have investigated cubosomes loaded with compounds derived from algae.

**Table 2:** DLS and ELS Measurements (Apparent Hydrodynamic Diameter (D_h_) of the Nanoparticles, Polydispersity Index (PdI), and ζ-potential of caulerpin@cub and Empty Cubosomes at 25 and 37 °C^a^

	T (°C)	caulerpin@cub	empty cubosomes
D_h_ (nm)	25	113 ± 2	118 ± 1
	37	115.0 ± 0.3	122 ± 2
PdI	25	0.20	0.23
	37	0.19	0.25
ζ-potential (mV)	25	–35 ± 1	–35 ± 1
	37	–31 ± 2	–32 ± 1

a Data are reported as average value ± SD.

No significant differences in size, polydispersity, or ζ-potential were observed, indicating that the temperature increase does not affect these parameters of the formulation, thereby confirming its stability for potential *in vivo* applications.

Concerning the long-term stability of the formulation (Figure S2), the apparent hydrodynamic diameter of the nanoparticles, especially up to week 7, did not show significant changes, while the PdI value, not exceeding 0.250, confirmed a narrow particle size distribution. The measured ζ-potential, with values always in the range of −30/–40 mV can be called into play for the observed colloidal stability.^63^

### 3.3 Biological Activity

#### 3.3.1 Anticancer Effect Evaluation

The antitumor effect (Figure 4) of caulerpin@cub was studied by a time-dependent MTT cytotoxicity assay on human pancreatic cancer cell lines (BxPC-3). The tests were performed at different caulerpin concentrations, 0.5, 1, 2, 3, and 4 μM corresponding to 56, 112, 223, 334, and 445 μg/mL of MO, after 4 incubation periods, 4, 8, 24, and 48 h (Figure 4A). The series of MTT tests were performed to choose the most efficient caulerpin concentration for further internalization and bioimaging experiments.

**Figure 4:**
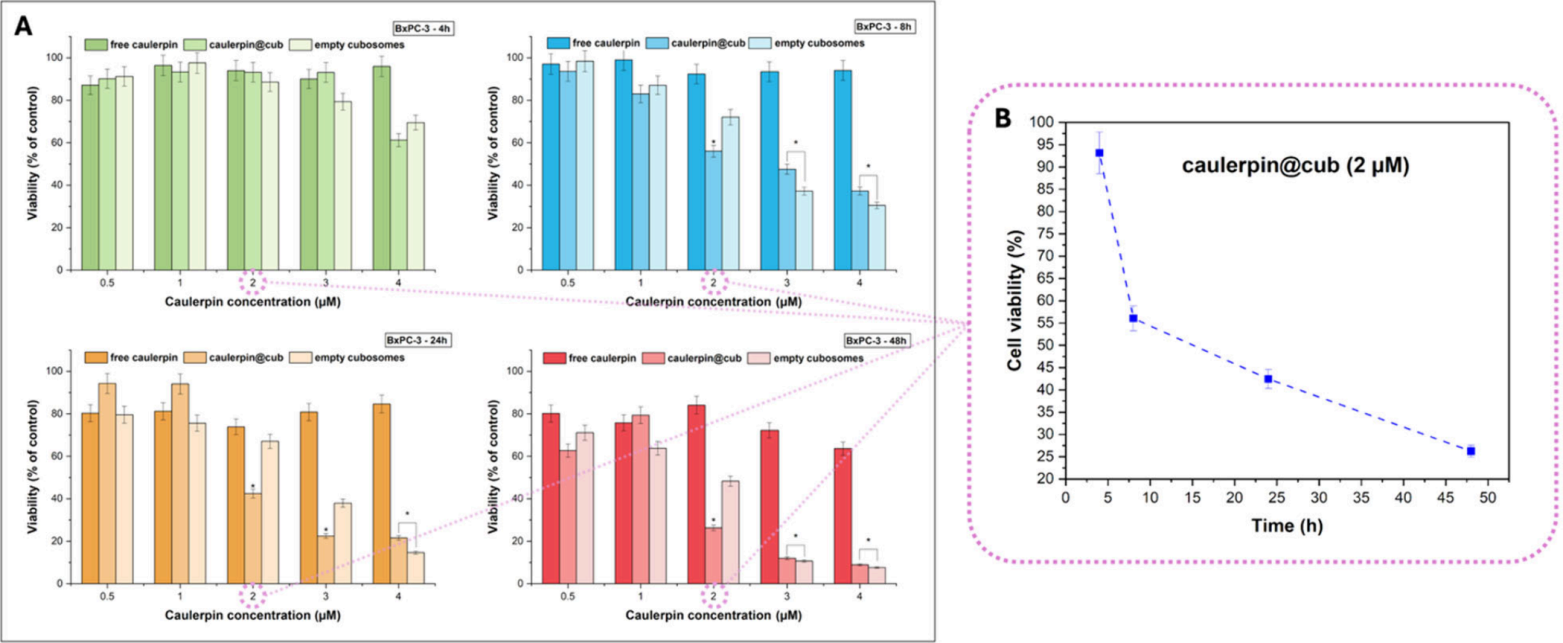
*In vitro* cytotoxicity experiments. MTT test on BxPC-3 cells after 4, 8, 24, and 48 h of incubation with free caulerpin, empty cubosomes, or caulerpin@cub at different alkaloid concentrations (0.5, 1, 2, 3, and 4 μM), corresponding to 56, 112, 223, 334, and 445 μg/mL MO (A); dependence of cell viability on incubation time for the selected concentration of 2 μM (B). *p ≤ 0.05.

After 4 h of incubation with samples at 4 μM caulerpin concentration, significant differences between samples can be observed. Particularly, caulerpin@cub induced about 60% reduction of the cell’s viability vs control. However, the empty cubosomes are weakly cytotoxic at this concentration as well as at 3 μM, indicating that these concentrations are too high. A lower alkaloid quantity is not toxic for BxPC-3 after 4 h of incubation with all samples. After 8 h of incubation, significant differences between the samples emerge starting at a concentration of 2 μM. At this concentration, caulerpin@cub exhibits higher cytotoxicity (approximately 55% cell viability vs control) compared to both empty cubosomes and the free compound. At higher concentrations, the empty cubosomes also demonstrate toxicity. MO, the lipid component used to form cubosomes, has been previously evaluated for cytotoxicity on various cell lines, with several studies reporting that significant cytotoxic effects often occur at concentrations of about 100 μg/mL.^53,64,65^ As caulerpin was diluted in the formulation to obtain the concentrations studied by the MTT assay series, the MO was also proportionally diluted (corresponding MO concentrations are specified in the previous paragraph). The results suggest that the cytotoxicity threshold of the unloaded nanocarriers in this study is even higher than previously reported, as the concentration of 112 μg/mL MO (equivalent to 1 μM of caulerpin) still presented over 60% cell viability after 48 h, indicating only “weak” cytotoxicity. This suggests an increased biocompatibility compared to conventional Pluronic-based cubosomes, as shown in our previous work.^55^ The concentration-dependent cytotoxicity becomes more pronounced at 48 h, particularly at higher MO levels (223, 334, and 445 μg/mL), which aligns with findings from several earlier studies indicating that such concentrations significantly surpass the biocompatibility limits for these nanocarriers.^53,64,65^ After 24 h, the difference becomes more pronounced, with caulerpin@cub at 2 μM showing strong cytotoxicity, reducing cell viability to about 40%. In contrast, free caulerpin does not exhibit significant toxicity after 24 h, highlighting the advantage of encapsulating caulerpin within cubosomes. Although, research on caulerpin cytotoxic effects against pancreatic cancer remains an unexplored area, caulerpin in its free form has been tested against many human cancer cell lines.^3,4,14,25^ For instance, IC_50_ after 48 h of incubation was demonstrated only at very high concentrations (119 and 179 μM) of caulerpin against colorectal cancer cell lines: HCT-116 and HT-29, respectively.^4^ This phenomenon may explain the lack of a strong cytotoxic effect (about 60% of remaining cell viability) in the case of free caulerpin tested in our study, even at the highest concentration of 4 μM and the longest incubation (48 h). After this time, caulerpin@cub at 2 μM reaches 25% (strong cytotoxicity); however, it can be observed that even the empty cubosomes start to be significantly cytotoxic to BxPC-3 (about 50% of remaining cell viability). Ferramosca et al. have investigated the cytotoxicity of caulerpin against two human ovarian carcinoma cell lines (2008, wild type, and C13, cisplatin-resistant type) after an even longer incubation time (72 h) and achieved only 60% of cell viability in the case of 2008, and just over 30% in the case of C13.^25^ This highlights the remarkable potential of caulerpin encapsulation in cubosomes for the treatment of pancreatic cancer as it significantly reduces the incubation time required to achieve substantial cytotoxicity, even against the highly resistant BxPC-3 cell line. The work of Zhou et al. describes studies on BxPC-3 cells and their resistant subtypes (BxPC-3-GR).^66^ The IC_50_ after 48 h of incubation with gemcitabine for BxPC-3 cells was 0.024 ± 0.003 μM, while for BxPC-3-GR cells it increased to 2.68 ± 0.104 μM, indicating a significant development of drug resistance in this type of pancreatic tumor cells.^66^ Furthermore, some other well-known anticancer drugs, namely, cisplatin, capecitabine, and irinotecan, presented an IC_50_ of 1.656 ± 0.284 μM, 7.890 ± 0.911 μM, and 2.530 ± 0.082 μM, respectively, after 48 h of treatment on BxPC-3 cells.^66^ In our work, even at the highest tested dose (4 μM), free caulerpin showed only 60% of cell viability after 48 h, whereas after loading in nanocarriers, significant cytotoxicity (approximately 40%) was demonstrated already after 24 h at a concentration of 2 μM. Such value is comparable with the IC_50_ of the mentioned pancreatic cancer drugs (cisplatin, irinotecan), almost 4 times lower than IC_50_ of capecitabine studied by Zhou et al. on BxPC-3 cells, as well as comparable to gemcitabine studied against resistant BxPC-3 populations. Remarkably, our result of 40% cell viability in cells treated with caulerpin@cub was achieved after only 24 h of incubation—half the duration used in comparable studies—indicating its strong cytotoxic effect relative to already known pancreatic cancer drugs. Mechanistically, caulerpin targets mitochondrial complex I, PTP1B, and the AMPK/mTORC1 axis,^14,24^ pathways distinct from gemcitabine’s nucleoside-analog mechanism.^67^ This orthogonal mode of action makes caulerpin@cub a potential candidate for overcoming gemcitabine resistance or combination therapy.

In summary, these results indicate that a concentration of 2 μM caulerpin was the most suitable for subsequent biological tests on BxPC-3 cells. This choice is supported by the observed influence of caulerpin encapsulation on antitumor bioactivity along with the demonstrated biocompatibility of the empty cubosomes. The optimal time of action is 24 h of incubation, because after 48 h, moderate cytotoxicity was observed also in the case of empty cubosomes. Figure 4B shows the dependence of cell viability on incubation time for the selected concentration of 2 μM in the case of caulerpin@cub, which decreases while extending the incubation time, highlighting the antitumor potential of the tested formulation.

#### 3.3.2 Bioimaging of the Cell Morphology

For comprehensive evaluation of the BxPC-3 cells morphology and thus the influence of the tested samples on the appearance and behavior of such cells, the assessment was based on three different microscopic experiments, showing both the entire cell culture on a larger scale by optical microscopy but also specific cells, cell clusters, or apoptotic features by live cell HTM or F-actin fluorescence bioimaging at a higher magnification.

##### 3.3.2.1 Optical Microscopy (Culture Morphology)

In order to assess the culture morphology of BxPC-3 pancreatic cancer cells, a series of images were taken supravitally by optical microscope after two incubation times: 8 and 24 h. Free caulerpin (2 μM), caulerpin@cub (2 μM), and empty cubosomes were examined against control cells (Figure 5). The images of BxPC-3 cells, shown on a scale of 500 μm, indicate significant differences in the morphology of human pancreatic tumor cells after incubation with selected samples. As for the control cells, in principle only the surface of their growth increases, the clusters are larger, and the condition of the cells is appropriate. Similar observations can be done by comparing the images of the empty cubosomes for 8 and 24 h, supporting their biocompatibility. On the other hand, cells treated with free caulerpin (2 μM), present smaller clusters or single small cells or their parts are visible, which indicate the cellular stress conditions and potential induction of apoptosis by caulerpin toward BxPC-3. Indeed, during apoptosis the cell actively breaks down its components and decreases in size by forming and separating portions of the cytoplasm and nucleus into apoptotic bodies.^68^ However, after longer incubation, apoptosis does not occur, and the cells continue to grow in small clusters, metabolizing caulerpin and regenerating in the process. In contrast to the above-mentioned samples, caulerpin@cub presents strong toxicity against BxPC-3, because in the image presenting cells treated with this sample after 24 h, only remnants of dead cells are visible. After the typical apoptotic process, cytoplasmic shrinkage, chromatin condensation, and nuclear fragmentation, as well as plasma membrane blebbing, apoptotic bodies are formed, which are phagocytosed and degraded.^69^ Therefore, in conclusion, these results indicate the potential effectiveness of caulerpin@cub in combating extremely resistant pancreatic cancer, additionally indicating the advantages of the encapsulation and biocompatibility of empty cubosomes.

**Figure 5:**
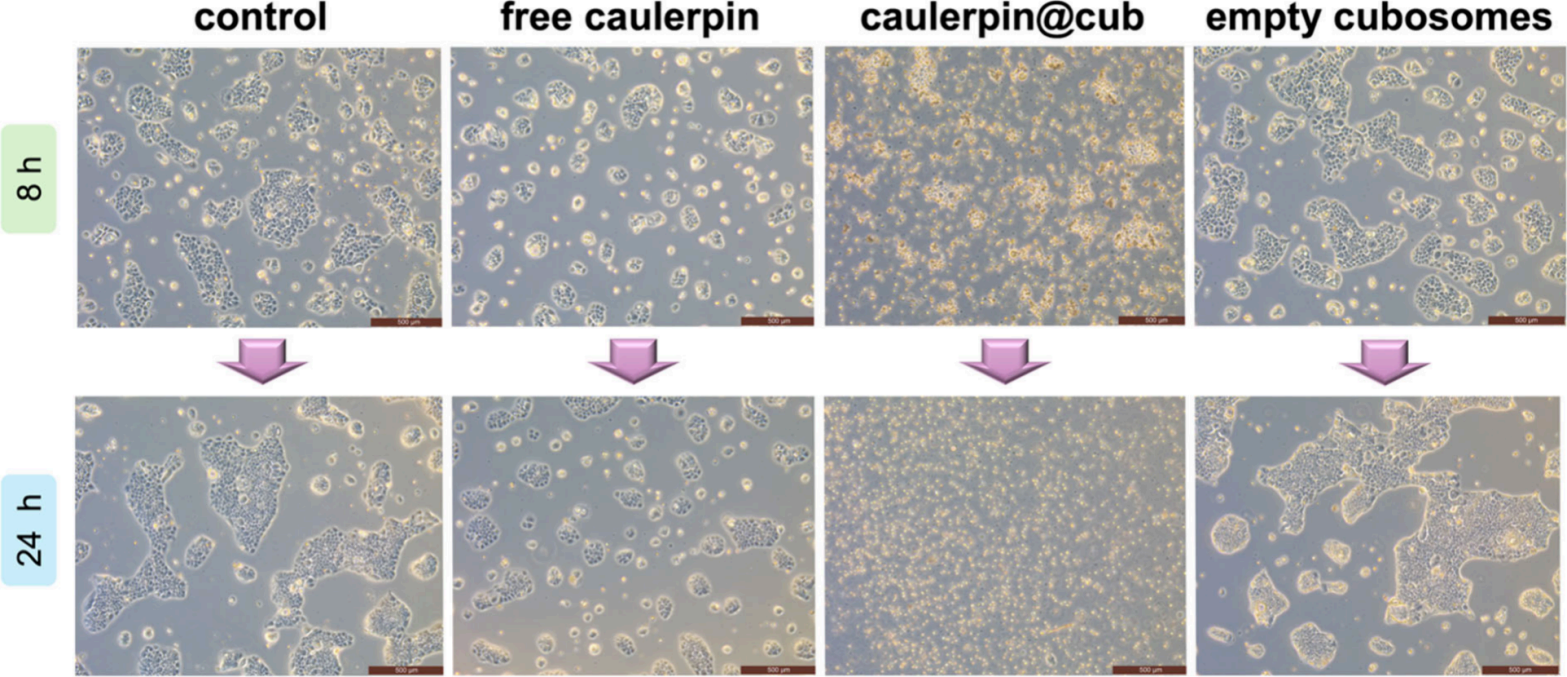
BxPC-3 cells morphology assessment by optical microscopy, after 8 and 24 h of incubation with different samples: free caulerpin (2 μM), caulerpin@cub (2 μM) and empty cubosomes, in comparison to untreated control cells. The scale bar represents 500 μm.

##### 3.3.2.2 Holotomographic Microscopy (HTM)

To go deeper into the details of the morphological changes in pancreatic cancer cells, a holotomographic microscope was used. This is a valuable new tool which combines two techniques, holography and tomography, useful for quickly distinguishing healthy from apoptotic cells, as well as monitoring both external and internal morphological cellular changes supravitally, using a non-invasive, label-free method.^70,71^ According to the studies of Salucci et al., HTM seems to be a reliable method for further analyzing well-established apoptotic features, including cell blebbing, chromatin condensation, micronuclei, and apoptotic bodies.^70^ BxPC-3 cells were treated with different samples: empty cubosomes, caulerpin@cub (2 μM), and free caulerpin (2 μM). Results relative to control cells are shown in Figure 6. The images were taken supravitally after 4, 8, and 24 h of incubation. Starting from the cells treated with free caulerpin, after 4 h the accumulation of lysosomes inside the cell is noticeable, indicating induced cellular stress and some processes related to autophagy, probably due to the presence of caulerpin. Autophagy, in addition to being a vital process for cellular turnover, is also induced by cellular stress conditions and serves as a crucial repair mechanism for damaged cells and helps them survive periods of temporary starvation.^72^ Lysosomes are membrane-bound organelles, present in all animal cells (except erythrocytes), within which the breakdown of materials taken up by endocytosis or autophagy, takes place.^73^ The extent of lysosomal destabilization during cellular stress conditions determines whether reparative autophagy, or typical cell death types, apoptosis or necrosis, will occur.^72^ Moreover, some vacuoles are visible there, another element indicating unfavorable and stressful conditions for cells. Vacuolization is one of the hallmarks of autophagy, which can further lead to necrosis or apoptosis.^69^ In cancer cells, under the influence of stress, e.g., exposure to anticancer drugs, lysosomes enlarge and fuse with autophagosomes, forming structures resembling vacuoles, such as doxorubicin-induced autophagolysosome formation in the resistant breast cancer cells.^74^ This phenomenon is consistent with previous reports, which indicate that the TFEB pathway is activated in response to stress, leading to increased lysosome biogenesis and autophagy.^75,76^ After the phase of intensive lysosomal fusion and the formation of large autophagolysosomes, cells can undergo a regeneration process, which is manifested by a decrease in the volume of these structures and restoration of homeostasis. Such reversible nature of lysosome reorganization suggests that they not only perform a degradative function but also actively participate in the adaptation and survival of cancer cells under stress conditions, e.g., drug stress.^76^ Additionally, Zhitomirsky and Assaraf emphasize the role of lysosomes in cancer cell resistance to chemotherapy, indicating that lysosomes sequester drugs, inactivating them and reducing the efficacy of treatment.^77^ In continuation of these findings, they showed that excessive accumulation of drugs in lysosomes can trigger lysosomal exocytosis—a process in which the contents of lysosomes, including stored drugs, are actively removed from cells, increasing their resistance to chemotherapy.^78^ In addition to this, in 2014, Maltese and Overmeyer have studied a nonapoptotic cell death called “methuosis”, with characteristic displacement of the cytoplasm by large, fluid-filled vacuoles that originate from macropinosomes.^79^ The vacuoles are particularly visible in the image of the caulerpin@cub sample. Cells treated with empty cubosomes after 4 h of incubation do not show any differences with the control, and only an enhanced number of intracellular vesicles is visible. After 8 h, cells incubated with free caulerpin exhibit morphological characteristics indicative of potential regeneration. This observation suggests that free caulerpin at a concentration of 2 μM is not toxic to BxPC-3 cells within this time frame. This result can be explained by the resistance of pancreatic cancer to most conventional treatment methods,^19^ due to its resilience related to remarkable capacity to orchestrate a special tumor protective microenvironment full of different components with huge impact on cancer cell biology, and therefore on a treatment resistance.^18,80^ One of the mechanisms of pancreatic cancer cells, described by Chand et al. as *hypoxia-induced resistance*, is linked to the nutrient- and oxygen-deprived tumor microenvironment. Hypoxia occurs when cancer cells do not receive enough oxygen, which is a result of uneven development of blood vessels within the tumor.^81^ This condition creates selective pressure that promotes the survival and growth of the most aggressive and resilient pancreatic cancer cells, making them highly resistant to cytotoxic chemotherapeutic agents.^17^ To overcome the harsh stress imposed by chronic hypoxia, pancreatic cells orchestrate a multifaceted response by activating hypoxia-inducible factors (HIFs),^82^ special protecting proteins able to regulate tumor cell biology. It turns out that this transcriptional response is involved in drug resistance, demonstrated by Cheng et al., who proved that under hypoxic conditions siRNA inhibition of either HIF-1α or NFκB, has sensitized pancreatic cells to gemcitabine, a commonly used cytostatic drug.^83^ When hypoxic cells were treated with gemcitabine at concentrations > 0.1 μM, their viability rate was significantly higher than that of normoxic. This suggests that hypoxia may contribute to chemoresistance in pancreatic cancer cells.^83^ Under normoxic conditions for 0.5 μM gemcitabine, the BxPC-3 cells viability was shown at the level of about 60% (comparable with 0.5 μM caulerpin@cub after 48 h), in contrast to hypoxia where this value was slightly below 80% after 48 h.^83^ However, for 1 μM gemcitabine, about 40% of viable cells were present (in normoxia) and about 70% (in hypoxia). In our study, a concentration of caulerpin twice as high was required to achieve a 40% cell viability when combined with nanocarriers; nevertheless, this effect was obtained in half the incubation time (24 h). Moreover, since caulerpin can additionally influence this process, by sensitizing cells to its action in hypoxia,^3,83^ this may constitute its additional advantage in the potential treatment of pancreatic tumor characterized by such conditions.

**Figure 6:**
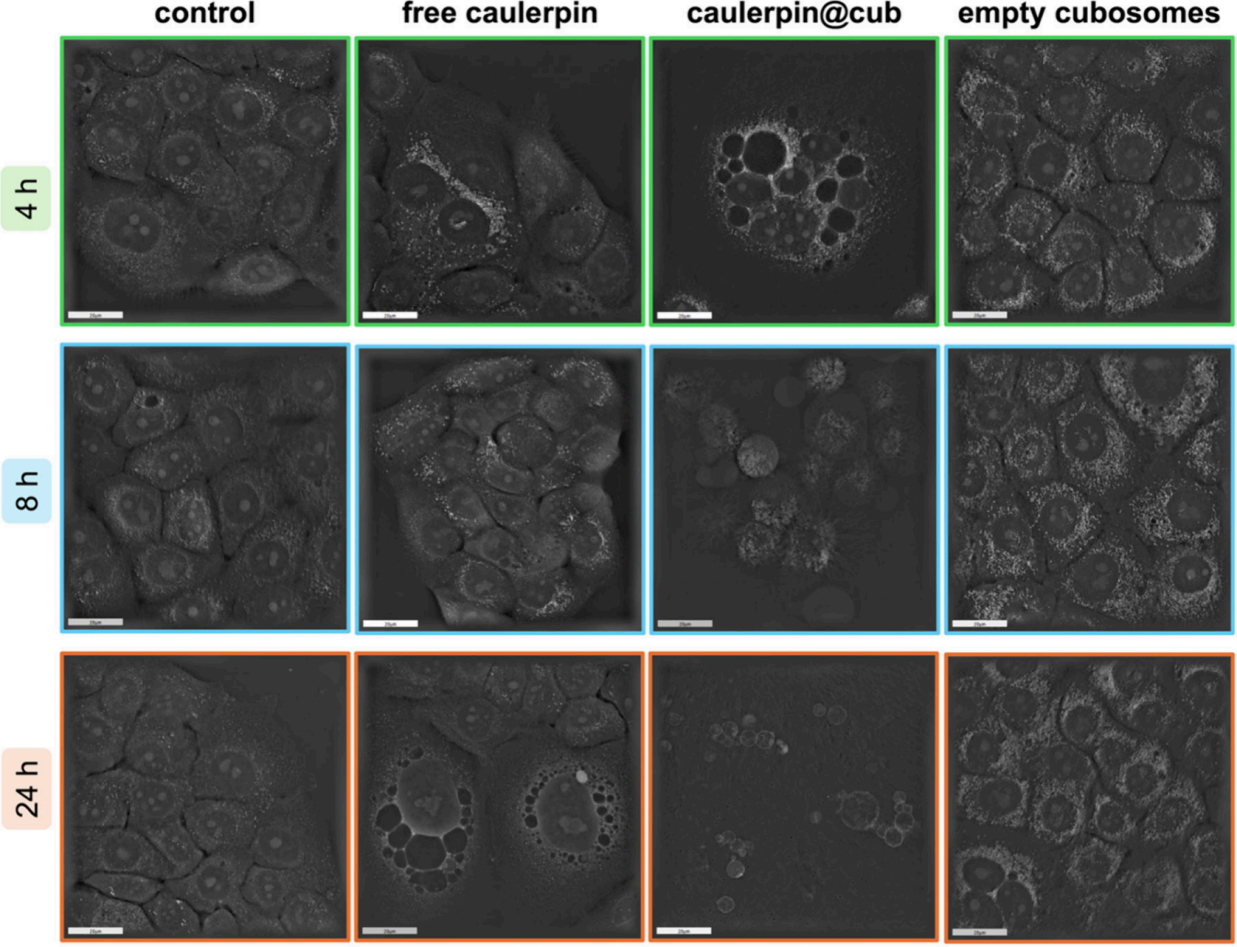
BxPC-3 cells morphology assessment by holotomographic microscopy, after 4, 8, and 24 h of incubation with different samples: free caulerpin (2 μM), caulerpin@cub (2 μM), and empty cubosomes, in comparison to untreated control cells. The scale bar represents 20 μm.

A significant difference with other samples can be observed in the image of cells treated with caulerpin@cub after 8 h of incubation because the process of apoptosis is operative. The round structures resembling blebs are the cytoplasm thrown out by cells that began to detach from the substrate and die. Dying cells are characterized by the formation of cellular buds, fragments, or blebs.^69^ Ziegler and Groscurth have observed the shrinkage of cell, blebbing, and apoptosis bodies formation as morphological features of ongoing cell death.^84^

According to Liu et al., caulerpin can disrupt mitochondrial ROS-regulated HIF-1 activation and HIF-1 downstream target gene expression by inhibiting the transport or delivery of electrons to complex III.^3^ As proved by Cheng et al. in 2014, this anticancer strategy based on overcoming the hypoxia-induced resistance mechanism may be responsible for sensitizing cancer cells to the action of potential drug.^83^ Another anticancer strategy may be related to caulerpin’s PTP1B inhibitory activity,^23,24^ since Xu et al. proved that inhibition of PTP1B protein blocks pancreatic cancer progression by targeting the PKM2/AMPK/mTOC1 pathway.^22^ Moreover, compared to the result of the free compound, a noticeable advantage of encapsulation is presented, probably by enabling the compound to reach the interior of cells and therefore increase its anticancer activity. These results show that although caulerpin may exhibit some anticancer properties, treating cells with this compound in its free form is insufficient to achieve the intended effect. After loading caulerpin in relevant nanocarriers, apart from introducing a stressful situation, an increase in apoptotic processes of cancer cells is visible, indicating a significant influence of the encapsulation on the caulerpin’s effectiveness in fighting pancreatic cancer. No significant premature release of caulerpin from the tested cubosomes, demonstrated by the *in vitro* drug release test (Figure S3), highlights the potential of its efficient delivery encapsulated within the nanocarriers with the protection from undesirable off-target activity or degradation, thus explaining its enhanced effect. After 8 h, the empty cubosomes induce the secretion of even more intracellular vesicles but do not fatally affect the condition of BxPC-3 cells, their morphology being similar to the control cells. After 24 h, free caulerpin begins to induce the formation of increasingly larger vacuoles, indicating still ongoing cellular stress. However, there are still many healthy cells that are visible. In the image of cells treated with caulerpin@cub after 24 h of incubation, basically only the remains of cells and thrown out cytoplasm suspended in the medium are visible (hence the poor sharpness of the image). After 24 h of cell incubation with empty cubosomes, the accumulation of vesicles is significantly greater but still not inducing cells’ death. These experiments show that the most effective against pancreatic cancer BxPC-3 cells is caulerpin (2 μM) loaded in cubosomes after 24 h. However, in the case of free caulerpin, the cells demonstrate the ability to metabolize it and regenerate over time, indicating a high level of resistance.

##### 3.3.2.3 Cytoskeleton F-Actin Fluorescence Staining

To confirm previously discussed results about the effectiveness of the given caulerpin@cub formulation against pancreatic cancer, we performed cytoskeleton bioimaging studies. Figure 7 presents the fluorescence bioimaging of BxPC-3 cells cytoskeleton by F-actin staining, treated with caulerpin, caulerpin@cub, and empty cubosomes formulations, along with control. Cells were incubated with test samples for 24 h at the 2 μM caulerpin concentration. It is important to notice that the results obtained with different imaging techniques may differ due to their unique properties and range of detection of cellular structures. While in the case of previous live HTM technique some structures are visible, after F-actin staining the cells are fixed, and the permeabilization may further remove the transient vacuoles visible by previous live cell bioimaging, so the outlines look smoother. Moreover, F-actin staining visualizes the actin cytoskeleton but does not reveal membranous structures like lysosomes or autophagolysosomes, with the intense peripheral actin signal potentially masking internal structures and limiting the ability to assess lysosomal quantity and function without additional specific staining.

**Figure 7:**
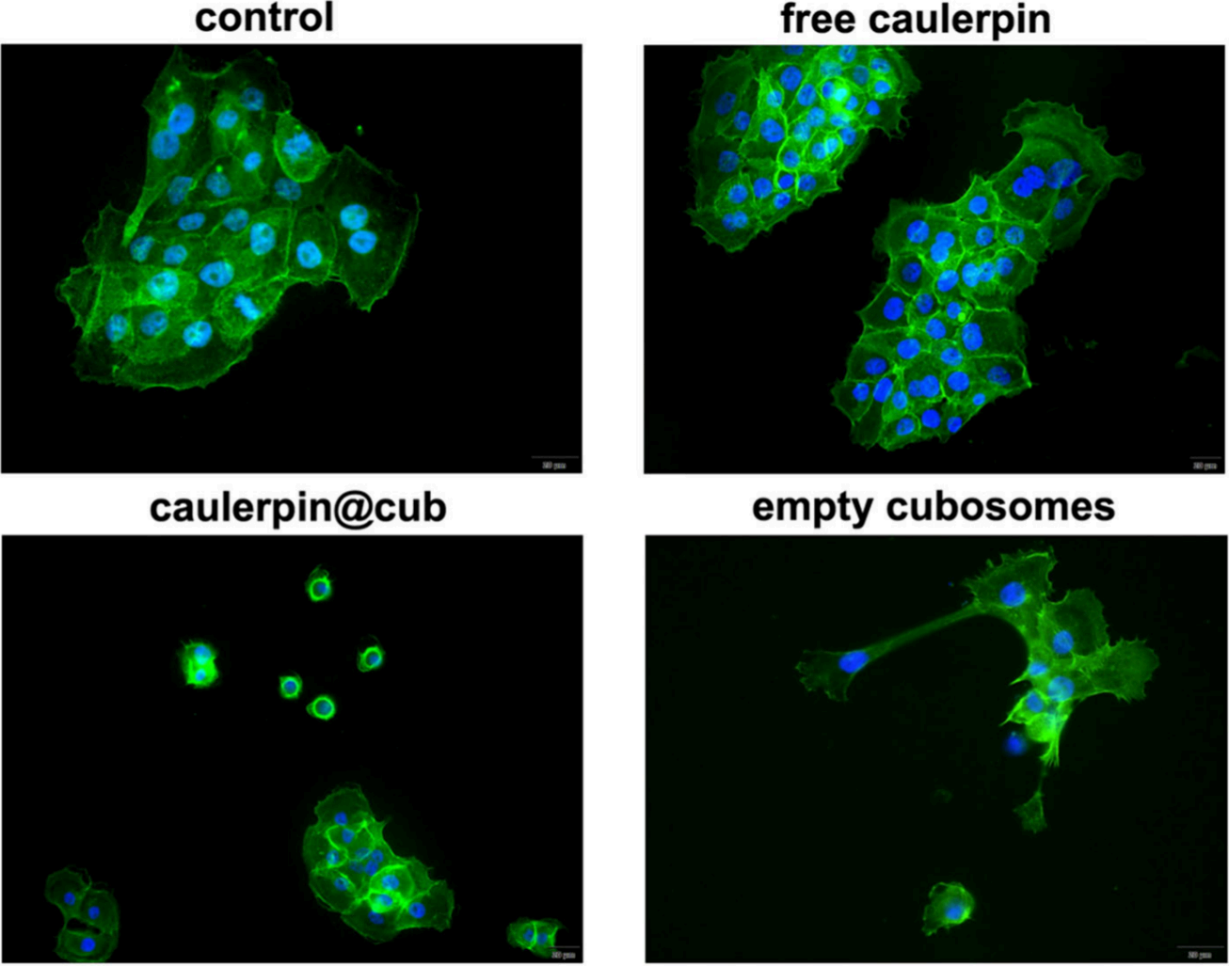
F-actin staining fluorescence bioimaging performed on human pancreatic cancer cell lines (BxPC-3) after 24 h of incubation. Cells were treated with different samples: free caulerpin (2 μM), caulerpin@cub (2 μM) and empty cubosomes, in comparison to untreated control cells. The scale bar represents 20 μm.

In the cell bioimaging treated with caulerpin@cub formulation, the disruption of intercellular connections can be observed, as well as the shrinkage of some of the BxPC-3 cells, and only the nuclei are visible with the residual cytoplasm, which was probably dehydrated in the process of apoptosis,^84,85^ as shown by previous HTM studies (section 3.3.2.2). In addition, there is a reduction in the nuclei size, probably related to nuclear condensation occurring during the process of cell death.^69^ This observation contrasts with the other images of the control cells, free compound (caulerpin), and the empty cubosomes, where the cells look healthy and have the appropriate morphology, considering the smaller number of clusters in the case of cells treated with cubosomes sample. Bioimaging of cells treated with free caulerpin reinforces the previous conclusion that BxPC-3 cells can metabolize the free compound and regenerate. Furthermore, when compared to caulerpin@cub, the benefits of caulerpin encapsulation—such as enhanced bioactivity efficiency—are once again demonstrated. This further illustrates that it is possible to overcome the challenges posed by the highly diverse and complex pancreatic tumor microenvironment, which often traps potential drugs and hinders their effectiveness. Thanks to the ability of cubosome nanocarriers to transport the bioactive cargo and reach the interior of cells, it is possible to induce cancer cells’ apoptosis. As previously mentioned, several pathways could explain such an anticancer activity of caulerpin. Available data reveal that caulerpin inhibits mitochondrial complex I, causing ΔΨm loss and an early ROS release.^14^ Furthermore, it was proved that further prolonged AMPK activation related to mTORC1-4E-BP1 axis inhibition, induced by caulerpin, disrupted glycolysis, and glucose metabolism in colorectal cancer cells, ultimately leading to their death.^14^ It was also demonstrated that caulerpin can inhibit PTP1B^24^ and block HIF-1 stabilization under hypoxia by inhibition of the mitochondrial electron transport to complex III.^3^ These mechanisms are involved in the intrinsic (mitochondrial) apoptotic pathway and match the lysosomal swelling, chromatin condensation, and membrane blebbing that we report. Similar conclusions were reached in earlier biological experiments presented in this work, further supporting the thesis that the formulation based on caulerpin encapsulated in cubic liquid crystalline nanocarriers at the appropriate concentration (2 μM) exhibits strong antitumor activity against BxPC-3 cells.

## 4 Conclusions

Pancreatic cancer remains one of the most challenging cancers to treat due to its high resistance to conventional therapies. With the aim to find an effective treatment method, this study explores the potential of caulerpin, a naturally occurring bisindole alkaloid derived from

*C. cylindracea*, which has demonstrated promising anticancer properties, but has not yet been tested against pancreatic cancer. The innovative approach presented here combines green chemistry with nanotechnology, namely, MAE to isolate pure caulerpin from *C. cylindracea* invasive algae biomass, and its encapsulation in novel biocompatible bicontinuous cubic liquid crystalline nanoparticles, cubosomes. The use of biocompatible surfactants (TC and Sp80) in the proposed formulation minimizes cytotoxicity, promoting efficient drug delivery with its protection against degradation, therefore increasing therapeutic potential. The physicochemical characteristics confirmed high encapsulation efficiency of the formulation, characterized by the presence of cubosomes coexisting with L3 sponge nanoparticles and vesicles. The formulation was proved stable even at 37 °C. A series of cytotoxicity tests allowed for the selection of the appropriate, optimal concentration of caulerpin (2 μM) for subsequent studies. After the initial assessment of cancer cells morphology by optical microscopy, an in-depth morphological analysis was performed using the HTM technique, suitable for the investigation of apoptotic features. Moreover, the anticancer effect of the caulerpin@cub formulation against BxPC-3 cells was confirmed by F-actin bioimaging, indicating, among others, a strong cell shrinkage and destruction of the cytoskeleton. In general, this work demonstrated significant apoptotic effects on human pancreatic cancer cells of the caulerpin encapsulated within cubosomes, showing superior antitumor activity, in contrast to the free compound. Loaded caulerpin is not metabolized by cancer cells as quickly as its free form, which enables its effective action by inducing apoptotic processes and ultimately leading to cells death. These results suggest the possibility of resistance problem reduction and a more effective anticancer action.

## Supplementary Material



## Notes

^1^H NMR spectrum of isolated caulerpin in CDCl_3_ along with its description, results of the long-term formulation stability test, as well as additional experimental data and results concerning the *in vitro* drug release test (PDF)
