# Supplementary material for: Caulerpin Delivery via Pluronic-Free Cubosomes: Unlocking the Therapeutic Potential of a Pigment from an Invasive Marine Algae
Source: Mol Pharm. 2025 Jul 1;22(8):4747–61. doi: 10.1021/acs.molpharmaceut.5c00340 (PMC12326354; doi:10.1021/acs.molpharmaceut.5c00340)
Supplement: Supplementary file 1 [file mp5c00340_si_001.pdf]

SUPPORTING INFORMATION

Caulerpin Delivery via Pluronic-Free Cubosomes: Unlocking the Therapeutic Potential of a Pigment from an Invasive Marine Algae

Karolina Krautforst,[1,2,3] Julita Kulbacka,[4,5] Marco Fornasier,[3,6] Rita Mocci,[1] Andrea Porcheddu,[1] Antonio Pusceddu,[7] Davide Moccia,[7] Sergio Murgia,[3,7]* Urszula Bazylińska[2]*

[1]*Department of Chemical and Geological Sciences, University of Cagliari, s.s. 554 bivio Sestu, I-09042 Monserrato, CA, Italy*

[2]*Department of Physical and Quantum Chemistry, Faculty of Chemistry, Wroclaw University of Science and Technology, Wybrzeze Wyspianskiego 27, 50-370 Wroclaw, Poland*

[3]*CSGI, Consorzio Interuniversitario per lo Sviluppo dei Sistemi a Grande Interfase, via della Lastruccia 3, 50019 Sesto Fiorentino, Florence, Italy*

[4]*Department of Molecular and Cellular Biology, Faculty of Pharmacy, Wroclaw Medical University, Borowska 211 A, 50-556 Wroclaw, Poland*

[5]*Department of Immunology and Bioelectrochemistry, State Research Institute Centre for Innovative Medicine Santariškių g. 5, LT-08406, Vilnius, Lithuania*

[6]*Division of Physical Chemistry, Department of Chemistry, Lund University, SE-22100 Lund, Sweden*

[7]*Department of Life and Environmental Sciences, University of Cagliari, Cittadella Universitaria Monserrato, S.P. 8 Km 0.700, I-09042 Monserrato, CA, Italy*

*Corresponding authors:

murgias@unica.it

urszula.bazylinska@pwr.edu.pl

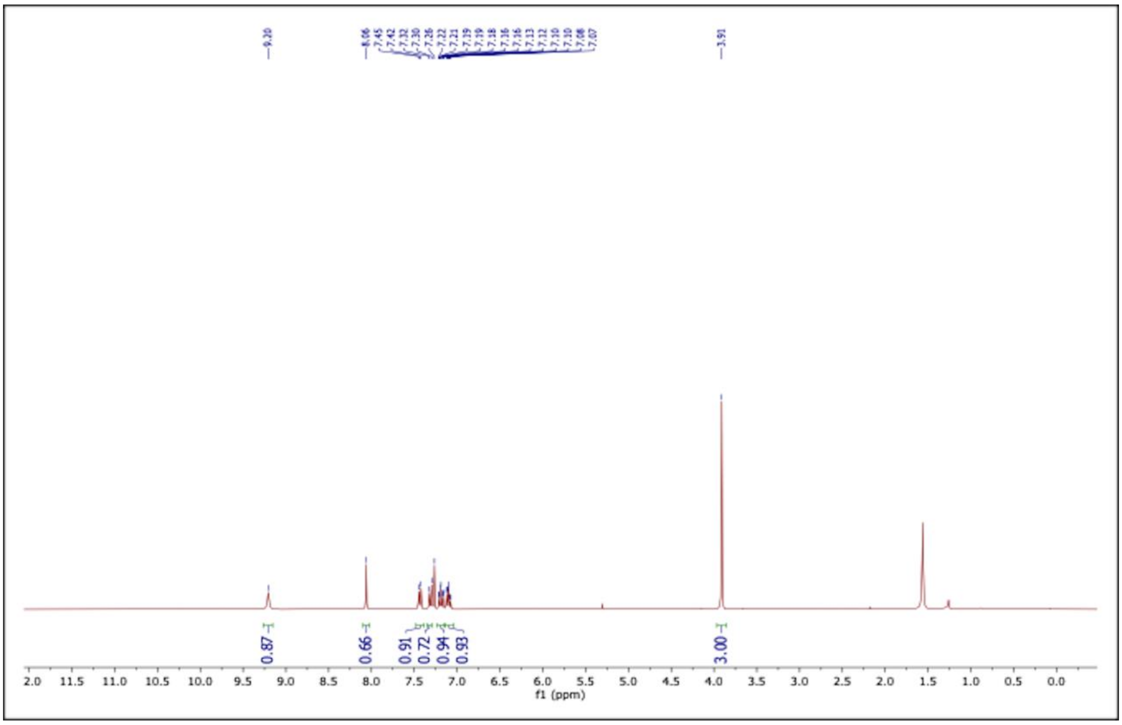

Figure S1. ^{1}H NMR spectrum of isolated caulerpin in $CDCl_3$

The ^{1}H NMR spectrum of the isolated compound presents the signals corresponding to caulerpin's chemical structure, correlating with the literature data of caulerpin standard in the same solvent.[1] The signal at 9.20 ppm is identified with 2 protons from 2 amino groups, hydrogen bonded to the ester carbonyls. Then, at 8.06 ppm, a signal from 2 protons bonded to the cyclooctatetraene ring connecting two indole groups is highlighted. Further, from 7.07 to 7.45 ppm, there are signals from 8 protons bonded to two aromatic rings and at 7.26 ppm, there is a signal from the solvent, $CDCl_3$. At 3.91 ppm, 6 protons from 2 methyl ester groups, bonded to the central ring, present the highest signal. The last peak is identified with the residual water. Overall, these spectroscopical characteristics suggest that the final product from *C. cylindracea* is indeed pure caulerpin.

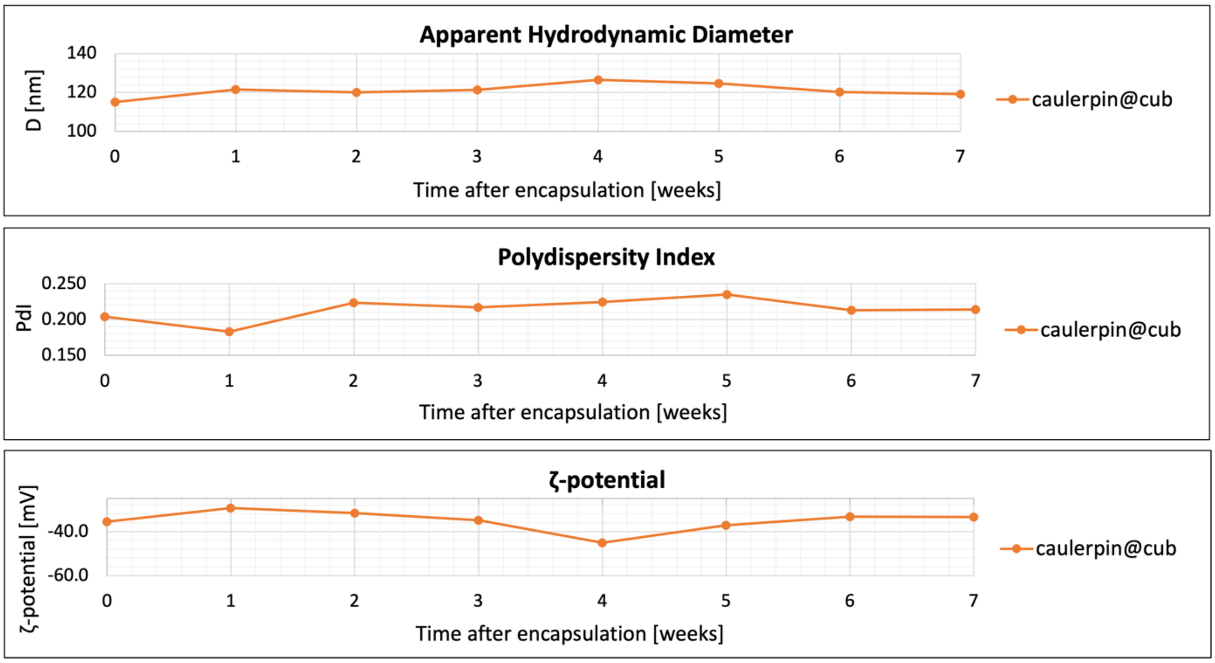

Figure S2. caulerpin@cub stability assessment by week-by-week DLS and ELS measurements.

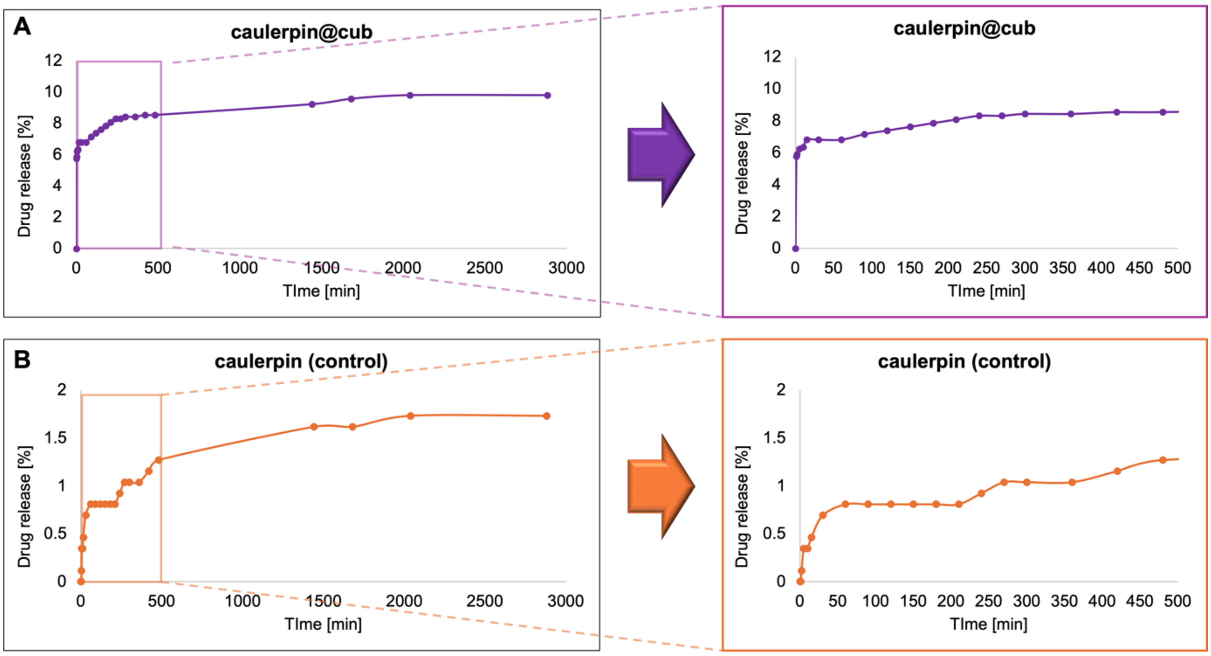

Figure S3. Encapsulated caulerpin release profiles (**A**) and the free pigment as a control (**B**).

Experimental

The release behavior of the encapsulated caulerpin was studied using UV-Vis spectroscopy combined with a dialysis technique according to the previous protocols[2–4]. A dialysis bag (Carl Roth, Germany) with a molecular weight cut-off of 14 kDa was used. Namely, 2 mL of the prepared cubosome formulation loaded with caulerpin was enclosed in the dialysis membrane and submerged in 25 mL of phosphate-buffered saline (PBS) at a physiological pH of 7.4, which acted as the release medium. The setup was then placed on a magnetic stirrer set to 200 rpm. To monitor drug release, 1 mL samples were taken from the external medium at regular time intervals and immediately replaced with fresh PBS of the same pH. The percentage of drug released was then calculated by comparing the amount of free caulerpin in the medium to the total drug content in the original sample using UV-Vis spectroscopy

Discussion

The drug release profile of caulerpin at physiological pH (7.4) from the cubosomes is reported in Figure SI3. The initial amount of caulerpin detected is related to non-encapsulated drug during cubosomes formulation. Therefore, release of caulerpin over 48 hours did not exceed 4%, indicating slow and low release of this potential drug. Hydrophobic drugs can be released from carriers into the aqueous environment due to diffusion across the carrier membrane, concentration differences, and drug-water interactions. Although these drugs are less likely to pass into water, they can gradually migrate out of the carrier, especially when the carrier

structure is destabilized or due to osmotic pressure. Such release is a natural physicochemical process that can occur despite the hydrophobic nature of the drug molecule.[4] This finding demonstrated that a small amount of free caulerpin may be released, despite its hydrophobic nature. Nevertheless, no significant premature release was observed during the test.

The drug is well encapsulated in the nanocarrier, as already proved by EE determination. Caulerpin is a hydrophobic pigment which is strongly bounded to the lipid matrix of the cubosome, thus hindering its diffusion into the aqueous environment of PBS. The premature release is avoided in this case, since it is expected that the nanocarrier will protect the drug from degradation or off-target activity before it reaches the target tumor tissue. The uptake of cubosomes by cancer cells is associated with the EPR (Enhanced Permeability and Retention) effect, which involves passive accumulation of these nanocarriers within the tumor due to the specific structure of its blood vessels characterized by leakage. The cubosomes can "leak" from the bloodstream into the tumor tissue thanks to their appropriate size to achieve this effect (~20–200 nm).[5,6] Lyotropic liquid crystalline nanoparticles, such as cubosomes, are known for their increased bioavailability and prolonged drug action due to its sustained release.[7–10] Longer circulation in the blood is associated with greater chance of reaching the tumor (especially with the EPR effect). Long retention of the drug in the nanocarrier extend its half-life and allow for longer action in the body, expected in case of cancer treatment. Since the cubosomes are stable at physiological pH, they do not degrade or disintegrate, therefore we believe that caulerpin will not be significantly released before the nanocarrier rich tumor cells, where will be internalized via endocytosis and due to specific tumor conditions (such as acidic microenvironment and lysosomal enzymes) the breakdown of the cubosomes lipid matrix and release the drug intracellularly will be promoted. Since the anticancer activity of caulerpin@cub was proved by series of *in vitro* tests on BxPC-3 cells, we believe that such reasoning provides potential application *in vivo*.

References

(1) *ChemSpider Database, CSID:4481120*. https://www.chemspider.com/Chemical-Structure.4481120.html (accessed 2025-02-16).

(2) Bazylińska, U.; Warszyński, P.; Wilk, K. A. Influence of PH upon in Vitro Sustained Dye-Release from Oil-Core Nanocapsules with Multilayer Shells. *Colloids Surf A Physicochem Eng Asp* **2012**, *413*, 266–272. https://doi.org/10.1016/j.colsurfa.2011.12.006.

(3) Bazylińska, U.; Skrzela, R.; Szczepanowicz, K.; Warszyński, P.; Wilk, K. A. Novel Approach to Long Sustained Multilayer Nanocapsules: Influence of Surfactant Head Groups and Polyelectrolyte Layer Number on the Release of Hydrophobic Compounds. *Soft Matter* **2011**, *7* (13), 6113–6124. https://doi.org/10.1039/c1sm05395g.

(4) Waglewska, E.; Pucek-Kaczmarek, A.; Bazylińska, U. Self-Assembled Bilosomes with Stimuli-Responsive Properties as Bioinspired Dual-Tunable Nanoplatform for PH/Temperature-Triggered Release of Hybrid Cargo. *Colloids Surf B Biointerfaces* **2022**, *215*. https://doi.org/10.1016/j.colsurfb.2022.112524.

(5) Hollis, C. P.; Weiss, H. L.; Leggas, M.; Evers, B. M.; Gemeinhart, R. A.; Li, T. Biodistribution and Bioimaging Studies of Hybrid Paclitaxel Nanocrystals: Lessons Learned of the EPR Effect and Image-Guided Drug Delivery. *Journal of Controlled Release* **2013**, *172* (1), 12–21. https://doi.org/10.1016/j.jconrel.2013.06.039.

(6) Pramanik, A.; Xu, Z.; Shamsuddin, S. H.; Khaled, Y. S.; Ingram, N.; Maisey, T.; Tomlinson, D.; Coletta, P. L.; Jayne, D.; Hughes, T. A.; Tyler, A. I. I.; Millner, P. A. Affimer Tagged Cubosomes: Targeting of Carcinoembryonic Antigen Expressing Colorectal Cancer Cells Using In Vitro and In Vivo Models. *ACS Appl Mater Interfaces* **2022**, *14* (9), 11078–11091. https://doi.org/10.1021/acsami.1c21655.

(7) Mezzenga, R.; Seddon, J. M.; Drummond, C. J.; Boyd, B. J.; Schröder-Turk, G. E.; Sagalowicz, L. Nature-Inspired Design and Application of Lipidic Lyotropic Liquid Crystals. *Advanced Materials* **2019**, *31* (35), 1900818. https://doi.org/10.1002/adma.201900818.

(8) Wei, Y.; Zhang, J.; Zheng, Y.; Gong, Y.; Fu, M.; Liu, C.; Xu, L.; Sun, C. C.; Gao, Y.; Qian, S. Cubosomes with Surface Cross-Linked Chitosan Exhibit Sustained Release and Bioavailability Enhancement for Vinpocetine. *RSC Adv* **2019**, *9* (11), 6287–6298. https://doi.org/10.1039/c8ra10302j.

(9) Murgia, S.; Biffi, S.; Mezzenga, R. Recent Advances of Non-Lamellar Lyotropic Liquid Crystalline Nanoparticles in Nanomedicine. *Curr Opin Colloid Interface Sci* **2020**, *48*, 28–39. https://doi.org/10.1016/j.cocis.2020.03.006.

(10) Yaghmur, A.; Mu, H. Recent Advances in Drug Delivery Applications of Cubosomes, Hexosomes, and Solid Lipid Nanoparticles. *Acta Pharm Sin B* **2021**, *11* (4), 871–885. https://doi.org/10.1016/j.apsb.2021.02.013.
